# Resident CD34-positive cells contribute to peri-endothelial cells and vascular morphogenesis in salivary gland after irradiation

**DOI:** 10.1007/s00702-020-02256-1

**Published:** 2020-10-06

**Authors:** Takashi I, Yuichiro Ueda, Philipp Wörsdörfer, Yoshinori Sumita, Izumi Asahina, Süleyman Ergün

**Affiliations:** 1 Institute of Anatomy and Cell Biology, University of Würzburg, Würzburg, Germany; 2 Unit of Translational Medicine, Department of Regenerative Oral Surgery, Nagasaki University Graduate School of Biomedical Sciences, Nagasaki, Japan; 3 Basic and Translational Research Center for Hard Tissue Disease, Nagasaki University Graduate School of Biomedical Sciences, Nagasaki, Japan

**Keywords:** Salivary gland, Xerostomia, Radiation, Resident CD34-positive cells, Mesenchymal stem cells

## Abstract

Salivary gland (SG) hypofunction is a common post-radiotherapy complication. Besides the parenchymal damage after irradiation (IR), there are also effects on mesenchymal stem cells (MSCs) which were shown to contribute to regeneration and repair of damaged tissues by differentiating into stromal cell types or releasing vesicles and soluble factors supporting the healing processes. However, there are no adequate reports about their roles during SG damage and regeneration so far. Using an irradiated SG mouse model, we performed certain immunostainings on tissue sections of submandibular glands at different time points after IR. Immunostaining for CD31 revealed that already one day after IR, vascular impairment was induced at the level of capillaries. In addition, the expression of CD44—a marker of acinar cells—diminished gradually after IR and, by 20 weeks, almost disappeared. In contrast, the number of CD34-positive cells significantly increased 4 weeks after IR and some of the CD34-positive cells were found to reside within the adventitia of arteries and veins. Laser confocal microscopic analyses revealed an accumulation of CD34-positive cells within the area of damaged capillaries where they were in close contact to the CD31-positive endothelial cells. At 4 weeks after IR, a fraction of the CD34-positive cells underwent differentiation into α-SMA-positive cells, which suggests that they may contribute to regeneration of smooth muscle cells and/or pericytes covering the small vessels from the outside. In conclusion, SG-resident CD34-positive cells represent a population of progenitors that could contribute to new vessel formation and/or remodeling of the pre-existing vessels after IR and thus, might be an important player during SG tissue healing.

**Electronic supplementary material:**

The online version of this article (10.1007/s00702-020-02256-1) contains supplementary material, which is available to authorized users.

## Introduction

The treatment for many patients with head and neck cancer is usually radiotherapy either alone or in combination with surgery and chemotherapy. After such treatment, although the patient’s prognosis is improved, radiation-induced damage to normal tissue leads to organ dysfunction, especially SG hypofunction. Despite many advances in modern high-precision radiotherapy techniques such as 3-dimensional conformal radiotherapy (3D-CRT) and intensity modulated radiation therapy (IMRT), the treatment still results in irreversible SG damage (e.g. xerostomia, dysphagia, dental caries, oropharyngeal infections, and oral mucositis (Seiwert and Cohen 2005; St John et al. 2006; Braam et al. 2006). Among these side effects, radiation-induced xerostomia is the most common complication of radiotherapy for head and neck cancer, significantly lowering the quality of life in the long-term survivors (Malouf et al. 2003; Trotti et al. 2003; Dirix et al. 2006). Therefore, various experimental approaches, such as the use of gene therapy, tissue engineering, and cell-based therapy, have been explored to develop strategies to functionally restore radiation damaged SGs (Baum et al. 2009; Sumita et al. 2011; Tanaka et al. 2018; I et al. 2019). However, there are currently no satisfying therapies for patients with such SG hypofunction.

Although salivary glands are basically composed of slowly proliferating highly differentiated cells, they are classified as exquisitely radiosensitive tissues like lymphoid organs, bone marrow, gonads and small intestines (Grundmann et al. 2009). In radiotherapy of patients suffering from head and neck cancers, it is known that irradiated glands exhibit distinct parenchymal loss accompanied by acinar atrophy, excessive progression of fibrosis and ductal dilatation, which leads to serious pathological remodeling of the glands and reduced saliva production. However, the detailed mechanism of radiation-induced xerostomia is still unclear (Radfar and Sirois 2003; Wu and Leung 2019).

In contrast, recent studies have revealed that tissue-resident stromal cells may contribute to regeneration and repair of damaged tissues. For instance, it has been shown that stromal cells play a supportive role in satellite cell-mediated skeletal muscle regeneration during eccentric contraction-induced skeletal muscle injury (Manetti et al. 2019). Moreover, joint-resident mesenchymal stromal cells support the repair of joint damage in osteoarthritis (McGonagle et al. 2017; Sanjurjo-Rodriguez et al. 2019) and CX3CR1^+^ synovial tissue-resident macrophages modulate inflammation during chronic inflammatory diseases such as rheumatoid arthritis (Culemann et al. 2019). Likewise, tissue-resident macrophages help to maintain tissue homeostasis and contribute to wound healing following injury in several tissues (e.g. skin, cardiac muscle, liver, skeletal muscle, and brain) (Rahmani et al. 2018; Dick et al. 2019; Santamaria-Barria et al. 2019; Kosmac et al. 2018; Hatton and Duncan 2019). One of the peripheral tissue niches that harbors different types of stem and progenitor cells is the vascular adventitia (Wörsdörfer et al. 2017). In this niche, vascular but also non-vascular stem and progenitor cells have been identified (Zengin et al. 2006; Klein et al. 2014; Wörsdörfer et al. 2017; Mekala et al. 2018). From the so-called “vasculogenic zone” of the vascular adventitia endothelial cells, smooth muscle cells and pericytes as well as antigen presenting cells such as macrophages and dendritic cells (Zengin et al. 2006; Psaltis et al. 2012; Psaltis and Simari 2015) were generated. More recently, a subpopulation of adventitial stem cells was shown to deliver spontaneously beating cardiomyocyte-like cells (Mekala et al. 2018). Only few studies focused on stromal cells in radiation damaged SGs so far, and their functional impact is not well understood. However, the morphological and functional analysis of irradiation-damaged SG stromal cells is an important prerequisite for the understanding of pathological processes leading to radiation-induced xerostomia and more important, the development of treatment strategies to functionally restore atrophic SG tissue.

The aim of this study was to determine the exact localization of CD34-positive resident stromal cells in the salivary gland and to follow their cellular fate after IR-induced damage of SG in a mouse model. We performed immunostainings on tissue sections of mouse SGs at different time points after IR and conducted statistical analyses based on the obtained results.

## Materials and methods

### Animals

C57BL/6JJcl mice (inbred strain) (CLEA Japan Inc., Tokyo, Japan) were used. All mice were kept under clean conventional conditions at the Nagasaki University animal center. All experimental procedures were performed in accordance with the guidelines approved by the Nagasaki University Ethics Committee (1605271307).

### Irradiation (IR)

Eight week-old female C57BL/6 mice were anesthetized with 10 μl/g body weight of ketamine (10 mg/ml) given by intraperitoneal (ip) injection and restrained in a container for IR. The submandibular glands were damaged by exposing them to a single dose of 12-Gy using gamma rays from PS-3100SB (Pony Industry Co, Ltd., Osaka, Japan). The radiation was collimated to the head and neck area to guarantee less than 10% beam strength in the rest of the body. After recovering from anesthesia, mice were returned to their cage and maintained in animal facility. At time of sacrifice (at 1, 3, 5 days, 1, 4, 8, and 20 weeks after IR), the mice submandibular glands were harvested.

### Salivary flow rate (SFR)

To measure the secretory function (salivary flow rate: SFR) of SGs, mice were kept under general anesthesia by injection of 10 μl/g body weight of ketamine. Whole saliva was collected after stimulation of secretion by subcutaneously administration of 0.5 mg/kg body-weight pilocarpine (Sigma Aldrich). Saliva was obtained from the oral cavity using a micropipette, and placed into pre-weighed 1.5 ml microcentrifuge tubes. Saliva was collected for a 10-min period and its volume was determined gravimetrically. SFR was determined at week 0, 4, 8, and 12 post-IR; *n* = 4 in each group at each time points.

### Histological and immunohistological analyses

The harvested submandibular glands were fixed in 4% paraform aldehyde (PFA) and embedded in paraffin. Five-micrometer sections were stained with hematoxylin and eosin (H&E) and examined microscopically at each time point. After deparaffinization and rehydration, stainings were performed. Immunofluorescence analyses were performed using rabbit anti-mouse CD31 antibody (1:50; Abcam), rat anti-mouse CD34 antibody (1:100; Santa Cruz Biotechnology), rat anti-mouse CD44 antibody (1:100; Biolegend), mouse anti-mouse α-SMA antibody (1:100; Abcam), goat anti-mouse Sca-1/Ly6 antibody (1:50; R&D Systems), rabbit anti-mouse c-Kit antibody (1:50; Abcam), mouse anti-mouse CD90 antibody (1:50; Sigma-Aldrich), and rabbit anti-mouse Ki-67 antibody (1:100; Abcam). Secondary Cy2-, Cy3- or Cy5-labeled antibodies were used to visualize primary antibodies. Sections were incubated with secondary antibodies and nuclei were stained with 4′,6-diamidino-2-phenylindole (DAPI) (Vector Laboratories). All antibodies were diluted in blocking solution. Immunofluorescence analyses detecting CD31, CD34, and CD44 were examined by fluorescence microscopy and the positive areas were analyzed by ImageJ software in a blinded manner under × 200 magnification (CD31; *n* = 4 at each time point, five different fields/*n* = 1, CD34; *n* = 4 at each time point, five different fields /*n* = 1, CD44; *n* = 4 at each time point, five different fields /*n* = 1). For immunohistochemical analyses, rat anti-mouse CD34 antibody (1:100; Santa Cruz Biotechnology), rabbit anti-mouse CEACAM1 antibody (1:100; Cell Signaling Technology), rabbit anti-mouse CD4 antibody (1:100; abcam) and rabbit anti-mouse Ki-67 antibody (1:100; Abcam) were used. Secondary biotin-coupled antibodies (Vector Laboratories) were used to detect the primary antibodies and the VECTASTAIN ABC Kit (Vector Laboratories) in combination with 3,3′-Diaminobenzidine (DAB) was used to visualize the secondary antibodies. Specimens were counterstained with hematoxylin or Nuclear Fast Red. Tissues were examined by light microscopy under × 200 magnification. The number of positive cells for CD4 and Ki-67 were counted in a blinded manner (CD4; *n* = 4, at each time point, ten different fields/*n* = 1, Ki-67; *n* = 2, at each time point, ten different fields/*n* = 1).

### Gene expressions analyses (*il-1β*, *tgf-β*, *pecam-1*, *cdh5*, *angptl4* and *aqp5*) in SG

Quantitative real time-PCR was used to determine the mRNA expression of *il-1β*, *tgf-β*, *pecam-1*, *cdh5*, *angptl4* and *aqp5* genes in submandibular glands; at 8-weeks post-IR (*n* = 3) compared to non-irradiated mice (*n* = 3). Total RNA was extracted using TRIzol reagent (Thermo Fisher Scientific) and first-strand complementary DNA synthesis was performed using the SuperScript First-Strand Synthesis System (Thermo Fisher Scientific). Complementary DNA was amplified using Takara-Taq DNA Polymerase (Takara). PCR reactions were performed with Mx3000P QPCR System (Agilent). The specific primer pairs used for quantitative RT-PCR are shown in Table 1. The primers were designed using Primer 3 software, version 4.0.0 (https://bioinfo.ut.ee/primer3/). Prior to use, the primer specificity was checked using the BLAST database at https://blast.ncbi.nlm.nih.gov/Blast.cgi. Glyceraldehyde-3-phosphate dehydrogenase (*gapdh*) was used as an internal control for quantitative real time-PCR.

**Table 1:** Mouse primer sets

Gene	Forward primer	Reverse primer
*il-iβ*	5´-GCTGAAAGCTCTCCACCTCA-3´	5´-AGGCCACAGGTATTTTGTCG-3´
*tgf-β*	5´-TTGCTTCAGCTCCACAGAGA-3´	5´-TGGTTGTAGAGGGCAAGGAC-3´
*aqp5*	5´-CCTTATCCATTGGCTTGTCG-3´	5´-CCCAGAAGACCCAGTGAGAG-3´
*cdh5*	5´-ACCGAGAGAAACAGGCTGAA-3´	5´-AGACGGGGAAGTTGTCATTG-3´
*angptl4*	5´-CCAGCTTAACAGCCTCCAAG-3´	5´-AAACTCCGTCAGTTGCTGCT-3´
*pecam1*	5´-TGCTCTCGAAGCCCAGTATT-3´	5´-TGTGAATGTTGCTGGGTCAT-3´
*gapdh*	5´-TGTGTCCGTCGTGGATCTGA-3´	5´-TTGCTGTTGAAGTCGCAGAG-3´

### Statistical analysis

All statistical analyses were carried out with JMP software (SAS Institute, Cary, NC). The normality of distribution was analyzed with the Shapiro–Wilk test. Student’s *t* test and the Mann–Whitney *U* test were conducted to compare two groups for parametric and non-parametric data, respectively. Experimental values are presented as mean ± SD; *p* < 0.05 was considered statistically significant.

## Results

### Histological observations in SG after IR

**Fig. 1:**
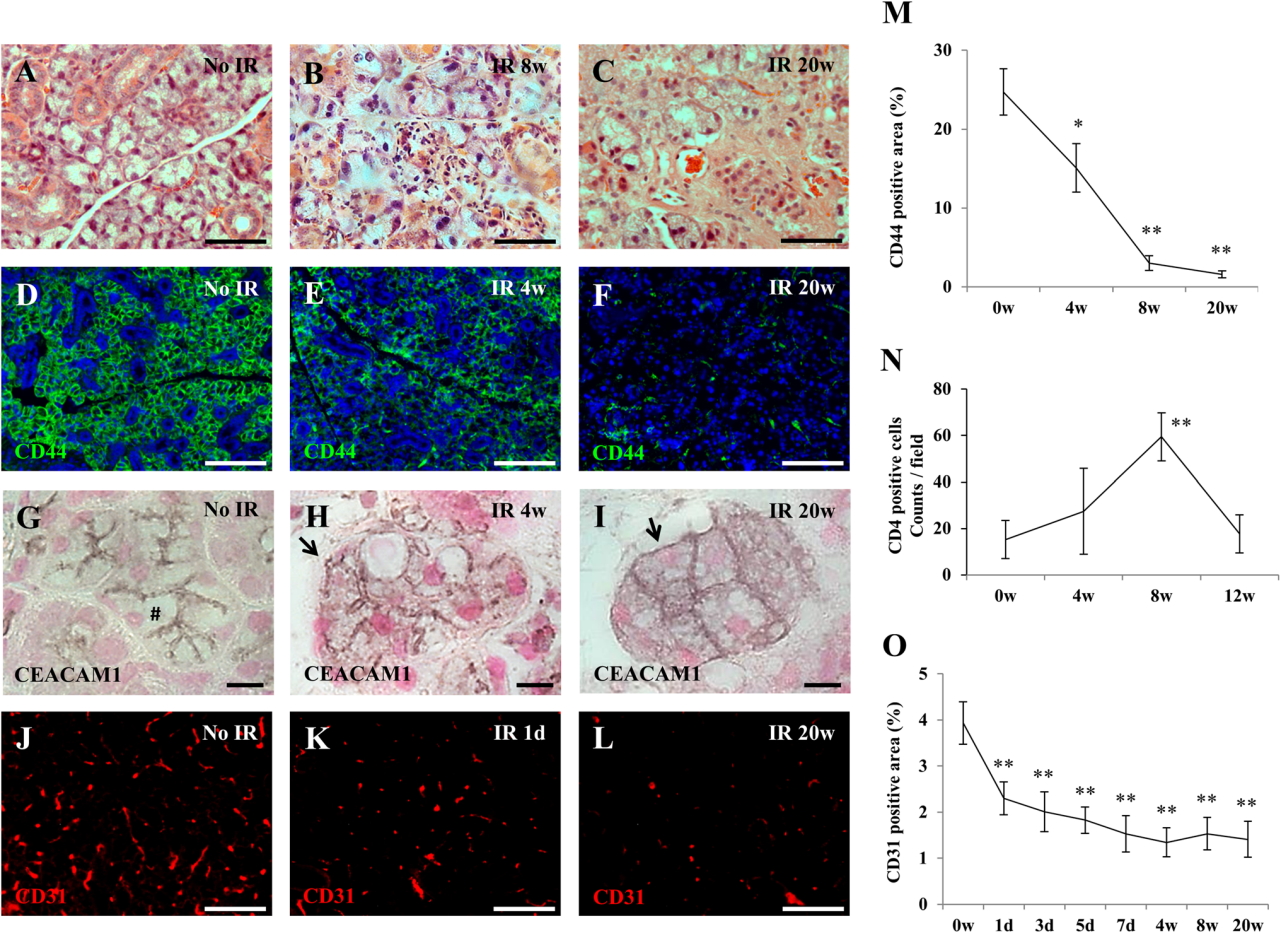
Histologic analysis of submandibular gland after IR. **a–c** Hematoxylin and eosin staining of non-irradiated submandibular glands (A), and SGs at 8-weeks (**b**), and at 20-weeks (**c**) after IR. Scale bar; 50 µm. **d–f** Immunofluorescence analyses of non-irradiated SGs (**d**), and SGs at 4-weeks (**e**), and at 20-weeks (**f**) after IR using antibodies targeted against CD44 (Green). Scale bar; 100 µm. Blue; DAPI, Green; CD44. **g–i** Paraffin-sections of submandibular glands with no IR (**g**), at 4-weeks (**h**), and at 20-weeks (**i**) after IR were stained for carcinoembryonic antigen-related cell adhesion molecule 1 (CEACAM1). Sections were counterstained with hematoxylin. CEACAM1 expression was observed only luminal (#) of acinar with no IR (**g**). In contrast, it was detectable at the whole acinar cell membrane, not luminal but also basal (arrow), at 4-weeks (**h**) and at 20-weeks (**i**) after IR. Scale bar; 10 µm. **j–l** Immunofluorescence staining for CD31 (Red) of submandibular glands with no IR (**j**), at 1-day (**k**), and 20-weeks (**l**) after IR. Scale bar; 50 µm. Red; CD31. **m** Changes of CD44-positive area (%) of salivary glands at 4-, 8-, and 20-weeks after IR. Asterisk represents statistical significance compared with no irradiated submandibular glands (**p < 0.01, *p < 0.05). **n** Changes of the number of CD4 positive cells at 4-, 8- and 12- weeks after IR. Asterisk represents statistical significance compared with no irradiated submandibular glands (**p < 0.01). **o** Changes of blood vessel area (%) in parenchyma of submandibular glands at 1-, 3-, 5-, 7-days, 4-, 8-, 12-, and 20-weeks after IR. Asterisk represents statistical significance compared with no irradiated submandibular glands (**p < 0.01)

To investigate the pathological changes after IR, we performed immunostainings on tissue sections of submandibular glands for different cell surface markers at different time points and quantified the obtained results. At 8-weeks after IR, CD4-positive inflammatory cells infiltrated the whole submandibular gland tissues (Fig. 1a–c, n) and mRNA expression of pro-inflammatory molecules (*il-1β* and *tgf-β* as well as molecules related to angiogenesis such as *pecam-1, cdh5,* and *angptl4* were significantly up-regulated (Suppl. Fig. 1). The number of acinar cells decreased gradually until 20-weeks after IR, and were replaced by fibrous tissue. This is evidenced by the fact that the positive area of CD44, which is expressed on the cell membrane of serous acini in SG (Maria et al. 2012), was decreased in irradiated mice at 4, (*p < 0.05), 8 and 20 weeks (**p < 0.01) post-IR compared to non-irradiated mice (Fig. 1d–f, m). Aquaporin-5 (AQP5), an exocrine gland-type water channel, is expressed in the salivary glands, in which it is mainly localized at the apical membrane of the acinar cells (Matsuzaki et al. 2012). At 8-weeks after IR, aqp5 mRNA expression was significantly down-regulated in submandibular glands compared to non-irradiated mice (Suppl. Fig. 1). In addition, the expression of CEACAM1 was studied as a marker that has been shown to be present at the luminal surface of several glandular and ductal epithelia (Nguyen et al. 2014; Takeyama et al. 2015; Weng et al. 2016). In contrast to non-radiated submandibular gland tissue, CEACAM1 immunostaining was detectable at the whole acinar cell membrane after IR, which indicates the loss of apical-basal polarity of the acinar epithelial cells (Fig. 1g–I). Furthermore, CD31-stainings revealed that the radiation damage caused a continuous reduction of capillaries in the submandibular gland parenchyma after IR (**p < 0.01). Of note, vascular impairment was already induced one day after IR (**p < 0.01) (Fig. 1j–l, o). Owing to these pathological changes, the secretory function of SGs (SFR) was severely reduced in irradiated mice after 4-weeks (Suppl. Fig. 2).

### Stable presence of resident CD34-positive cells after IR and their localization in SG

**Fig. 2:**
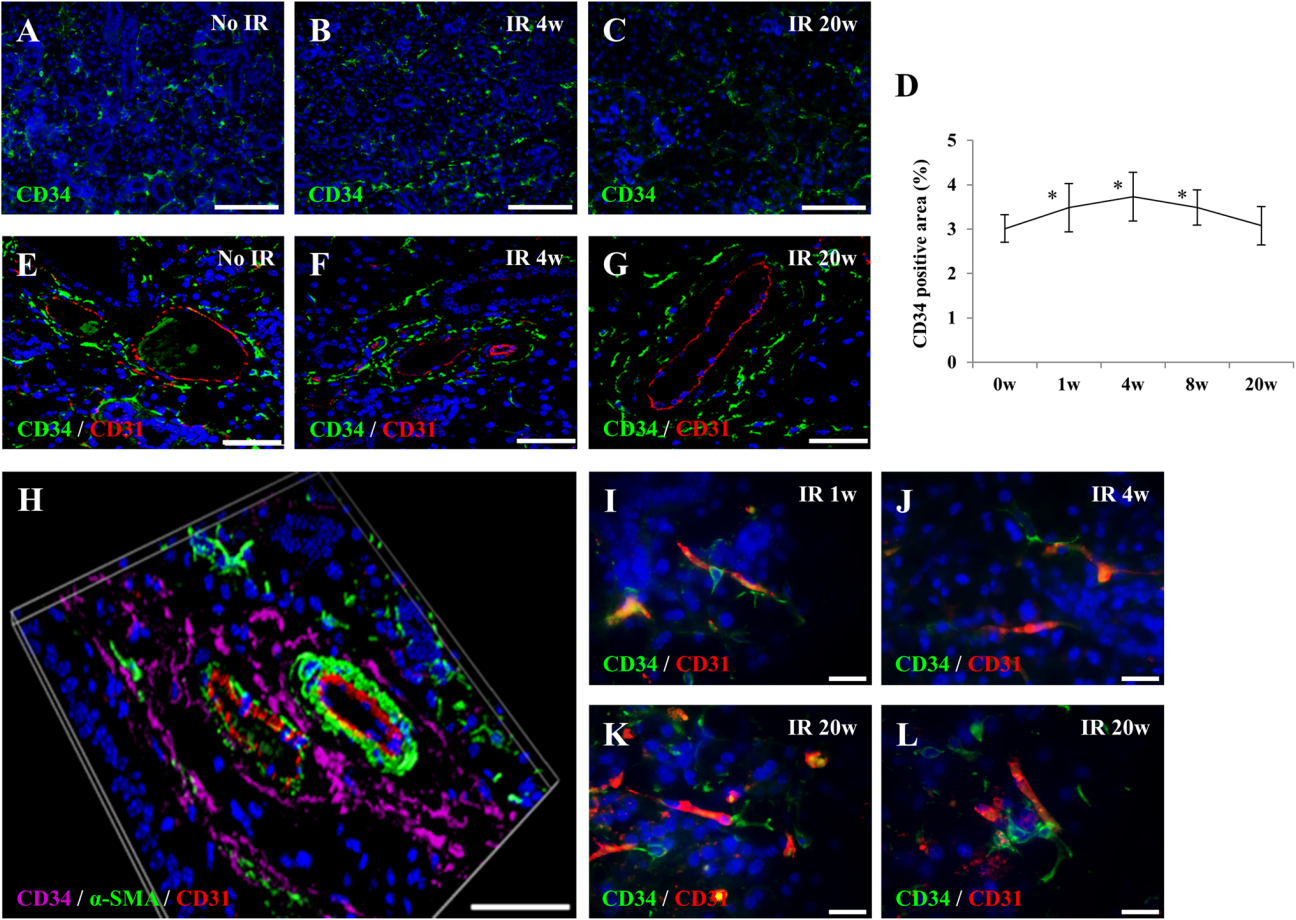
Localization of resident CD34-positive cells in submandibular glands. **a**–**c** Immunofluorescence staining for CD34 (Green) in parenchyma of submandibular glands with no IR (**a**), at 4-weeks (**b**), and at 20-weeks (**c**) after IR. Scale bar; 50 µm. Blue; DAPI, Green; CD34. **d** Changes of CD34-positive area (%) in parenchyma of submandibular glands at 1-, 4-, 8-, and 20-weeks after IR. Asterisk represents statistical significance compared with no irradiated submandibular glands (*p < 0.05). **e–g** Double immunofluorescence staining for CD34 (Green) and CD31 (Red) in submandibular glands with no IR (**e**) and at 4-weeks (**f**), and 20-weeks (**g**) after IR. Scale bar; 50 µm. Blue; DAPI, Green; CD34, Red; CD31. **h** Triple immunofluorescence staining for CD34 (Purple), α-SMA (α-smooth muscle actin) (Green), and CD31 (Red) in submandibular glands with no IR. Scale bar; 50 µm. Blue; DAPI, Purple; CD34, Green; α-SMA, Red; CD31. **i–l** Double immunofluorescence staining for CD34 (Green) and CD31 (Red) in parenchymal of submandibular glands at 1-week (**i**), at 4-weeks (**j**), and 20-weeks (**k**, **l**) after IR. Scale bar; 10 µm. Blue; DAPI, Green; CD34, Red; CD31

Tissue-resident CD34-positive cells were observed in the parenchyma of submandibular glands with/without IR (Fig. 2a–c and Suppl. Fig. 2). Their positive area significantly increased at week 1, 4, and 8 (*p < 0.05) post IR when compared to non-irradiated mice (Fig. 2d). Most resident CD34-positive cells clustered around blood vessels (Fig. 2e–g and Suppl. Fig. 2). Larger arteries, composed of intima (CD31-positive cells), media (α-SMA) and adventitia showed resident CD34-positive cells located within the adventitial layer of the vessel wall (Fig. 2h). Furthermore, some resident CD34-positive cells reached out to the tip of small capillaries directly contacting the endothelial cells (Fig. 2i–l).

### Resident CD34-positive cells support the maturation of blood vessels after IR

**Fig. 3:**
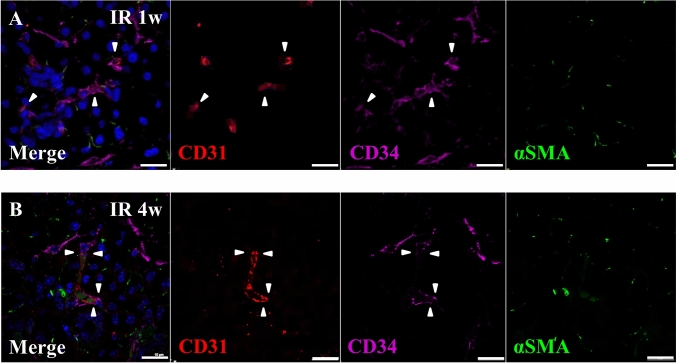
Resident CD34-positive cells accumulated around damaged blood vessels. **a**, **b** Triple immunofluorescence staining for CD31 (Red), CD34 (Purple) and α-SMA (α-smooth muscle actin) (Green) in parenchyma of submandibular glands at 1-week (**a**) and 4-weeks (**b**) after IR. Scale bar; 20 µm. Blue; DAPI, Red; CD31, Purple; CD34, Green; α-SMA

**Fig. 4:**
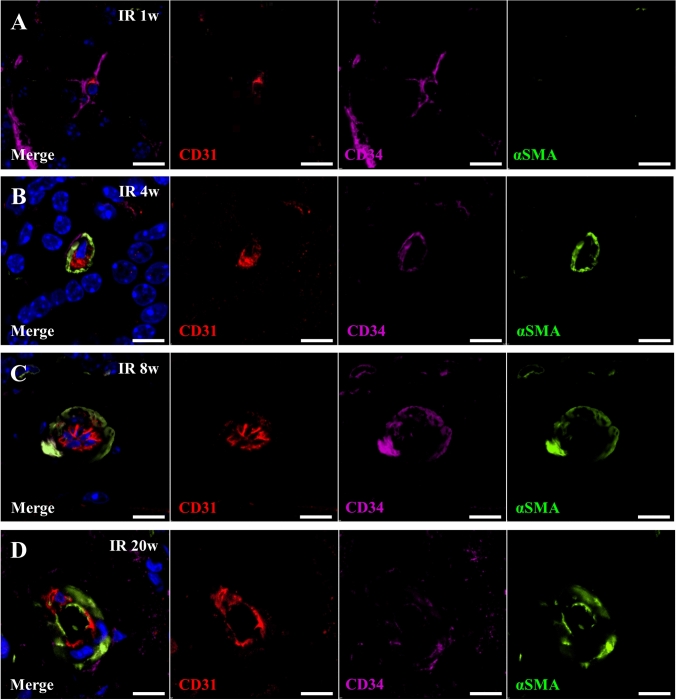
Resident CD34-positive cells have capacity of differentiation into smooth muscle cells**. a–d** Triple immunofluorescence staining for CD31 (Red), CD34 (Purple) and α-SMA (α-smooth muscle actin) (Green) in submandibular gland at 1-week (**a**), 4-weeks (**b**), 8-weeks (**c**), and 20-weeks (**d**) after IR. Scale bar; 10 µm. Blue; DAPI, Green; α-SMA, Purple; CD34, Red; CD31

We observed that SG-resident CD34-positive cells accumulated around radiation-impaired capillaries in the SG parenchyma at 1-week after IR (Fig. 3a). They were observed to be in close contact with the capillaries at 4-weeks after IR (Fig. 3b). In capillary cross-section of submandibular glands at 1-week after IR, resident CD34-positive cells enwrapped CD31-positive endothelial tubes from the outside (Fig. 4a) and exhibited co-immunostaining for α-SMA at 4-weeks after IR (Fig. 4b). Remarkably, at 8-weeks after IR, resident CD34-positive cells, which surrounded the endothelial layer of blood vessels, stained also strongly positive for α-SMA (Fig. 4c). At 20-weeks after IR, only weak expression of CD34 but a strong expression of α-SMA was detected which supports the assumption that a differentiation of CD34-positive cells towards a smooth muscle cell/pericyte phenotype (Fig. 4d) could have taken place.

Taken together, only few resident CD34-positive cells exhibited co-immunostaining for α-SMA in non-irradiated submandibular glands or 1-week after IR (Suppl. Fig. 4a, b). At 4- and 8-weeks after IR, most resident CD34-positive cells contacting small capillaries were also positive for α-SMA (Suppl. Fig. 4c, d). In contrast, their expression of CD34 decreased at 20-weeks after IR (Suppl. Figs. 4e, 2d).

### The immune phenotype of resident CD34-positive cells in SG

**Fig. 5:**
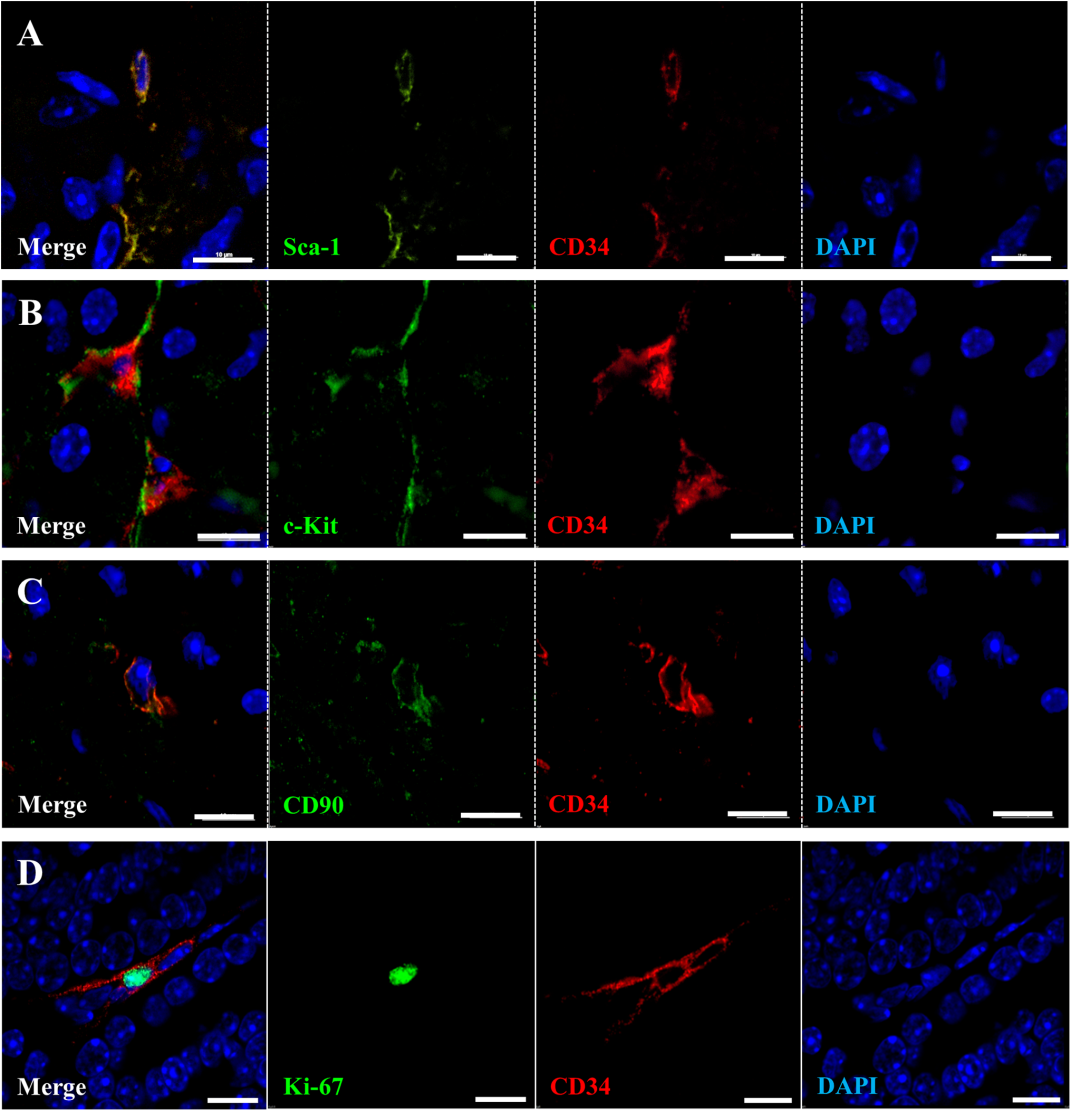
Characteristic analysis of resident CD34-positive cells in submandibular glands. **a–c** Double immunofluorescence staining for CD34 (Red) and Sca-1 (Green) (**a**), c-Kit (Green) (**b**), CD90 (Green) (C) in parenchymal of submandibular gland with no IR. Scale bar; 10 µm. Blue; DAPI, Green; Sca-1, c-Kit, and CD90, Red; CD34. **d** Double immunofluorescence for CD34 (Red) and Ki-67 (Green), in parenchymal of submandibular gland at 7 days after IR. Scale bar; 10 µm. Blue; DAPI, Green; Ki-67, Red; CD34

To characterize the immune phenotype of resident CD34-positive cells in submandibular glands, co-localization with marker proteins that are known to be preferentially expressed in MSC such as Sca-1, c-Kit, and CD90 was assessed. Double immunofluorescence revealed that some resident CD34-positive cells were also positive for Sca-1, c-Kit, and CD90 (Fig. 5a–c). The number of Ki-67-positive cells was decreased temporarily at 1- and 3-days (**p < 0.01) after IR. Thereafter, their number increased (Suppl. Fig. 5) and some of the CD34-positive cells did also show Ki-67 expression (Fig. 5d) at 7-days post IR. In contrast, they were negative for CD31 (endothelial cells) (Suppl. Fig. 6). These results indicate that a major portion of SG-resident CD34-positive cells shows an MSC-like phenotype. However, there is no considerable differentiation of SG-resident CD34-positive cells into endothelial cells.

## Discussion

This study demonstrates that SG-resident CD34-positive cells have the capability to contribute to morphogenesis of new vessels and stroma formation in SG after IR in mice. Briefly: (i) IR induces a considerable degeneration of SG tissue indicated by a loss of acinar cells and a reduced blood capillary density while SG-resident CD34-positive cells stably survive within the gland parenchyma and the adventitia of SG blood vessels, (ii) The number of SG-resident CD34-positive cells increases after IR and some CD34-positive cells cover the endothelial lining of newly forming capillaries from the outside, and (iii) SG-resident CD34-positive cells exhibit a MSC-like immunophenotype and differentiate into α-SMA positive cells, probably smooth muscle cells or pericytes. Thus, they might contribute to repair and regeneration of small blood vessels including capillaries. This suggests that SG-resident CD34-positive cells may serve as a source of progenitors that support morphogenesis, stabilization and maturation of blood vessels formed by postnatal vasculogenesis following IR.

Radiotherapy for head and neck cancers is one of the established therapeutic options. According to several previous studies it leads to side effects such as apoptosis of acinar cells and their loss that in turn results in reduction of saliva production and thus xerostomia. However, the detailed mechanisms of radiation-induced xerostomia remain unclear (Lim et al. 2013; Zhang et al. 2014). Since CD44 has been shown to be expressed in acinar epithelial cells of SG, we performed immunostaining for CD44 to assess severity of acinar cell damage as an indicative parameter for SG hypofunction after IR. CD44 is a transmembrane glycoprotein mediating cell-to-cell and cell-to-extracellular matrix adhesion that is involved in the preservation of the three-dimensional structure of tissues by binding to specific ligands, such as osteopontin, collagen, and chondroitin (Cichy and Puré 2003; Bourguignon et al. 2014). The most specific ligand for CD44 is hyaluronic acid that is an extracellular matrix polysaccharide deposited in the tissue stroma of different organs (Isacke and Yarwood 2002; Ponta et al. 2003). For many tissues CD44 is described as one of the markers for MSCs. However, in submandibular and parotid glands, CD44-positive cells show trans-epithelial electrical resistance, form tight junction structures under the transmission electron microscope (TEM) and secrete amylase which demonstrates that CD44 is a marker for serous acinar cells in SG (Maria et al. 2011). In this study, the CD44 positive area in SG gradually decreased at 4-weeks (61.07%), 8-weeks (12.26%), and 20-weeks (6.41%) after IR when compared with normal mice. Focusing on surviving acinar cells after IR, an excessive expression of CEACAM1 that indicates the loss of the apical-basal polarity was observed. CEACAM1 is a highly glycosylated cell adhesion molecule that belongs to the immunoglobulin super family and is involved in intracellular signaling cascades during several biological processes such endothelial barrier regulation, angiogenesis and vascular morphogenesis as well as tumor growth and immune regulation (Rueckschloss et al. 2016). Recently, the expression of CEACAM1 was shown to positively correlate with the expression of factors that are related to epithelial-mesenchymal transition (EMT) (Yoshikawa et al. 2017). During pathological fibrosis following radiation therapy, healthy salivary acinar cells undergo a partial EMT and differentiate into a fibroblast-like phenotype (Hall et al. 2010; Sisto et al. 2018). Therefore, the extension of CEACAM1 membrane localization to the basal side of surviving acinar epithelial cells could indicate, on the one hand, an increased interaction of these cells with the extracellular matrix (ECM) but on the other hand also an EMT process induced by IR. Radiation directly leads to vascular damage and a recent study on microvascular endothelial cells of SG in mice showed that a single radiation dose is enough to cause a significant reduction in capillary density (Mizrachi et al. 2016). Consistent with this report, our results show that a single radiation with 12 Gy induced severe capillary reduction in submandibular glands starting already 1 day after IR. Taking together, these data show that IR causes both, a loss of acinar cells and vascular impairment resulting in a severe, irreversible dysfunction of SG.

We asked the question, if a therapeutic dose of irradiation negatively impact the entire regenerative capacity of SG tissue? Here we show, that SG-resident CD34-positive cells stably survive within the parenchyma and the adventitia of blood vessels although IR induced severe pathological abnormality in SG as mentioned above. Over the last decades, the mature vessel wall has been identified as a niche for a variety of stem and progenitor cell populations (Alessandri et al. 2001; Tintut et al. 2003; Ingram et al. 2005; Zengin et al. 2006; Passman et al. 2008; Campagnolo et al. 2010; Psaltis et al. 2011; Klein et al. 2010, 2014; Mekala et al. 2018). In vitro analyses using embryonic aorta revealed that CD34^+^CD31^−　^cells in the aortic adventitia contribute to new vessel formation (Alessandri et al. 2001). Previously, we demonstrated the presence of such progenitors with CD34^+^KDR^+^CD31^−^ immunophenotype in the adventitia of adult human vessels using human internal thoracic artery (HITA) that give all vascular cell types including endothelial cells and more remarkably to some non-vascular cell types such as macrophages (Zengin et al. 2006).

Recently, it was reported that a SG-resident CD34-positive cell population contains a sub-population that could deliver MSCs with multi-lineage differentiation capability under in vitro culture conditions. Moreover, RNA sequence profiling of SG-resident CD34-positive cells exhibited elevated expression of genes encoding ERK, FGF/PDGF and Wnt signaling pathways that were shown to play key roles in the maintenance of homeostasis and regulation of organ regeneration (Togarrati et al. 2017). Furthermore, another recent study reported that CD34-positive cells which were found to be resident in minor SG were almost absent in patients who were suffering from severe primary Sjögren's syndrome indicating a potential role of this progenitor cell population in the control of local tissue homeostasis (Alunno et al. 2015). However, little is known until now about the fate and functions of SG-resident CD34-positive cells in situ after injury. We and others previously reported that CD34-positive adventitial stem cells have the capacity to differentiate into pericytes and smooth muscle cells in vitro and in vivo and contribute to new vessel formation (Invernici et al. 2007; Klein et al. 2011; Kramann et al. 2016; Campagnolo et al. 2010). As far as we are aware, our results demonstrate for the first time that SG-resident vascular wall-resident CD34-positive cells survive after IR and directly interact with radiation-injured and newly forming capillaries during the post-IR period enwrapping endothelial cells from the outside and differentiating into α-SMA positive cells. These could be pericytes and/or smooth muscles cells that are essential for vascular morphogenesis and stabilization. Of note, additional α-SMA positive cells were observed lining the acini or being located within the connective tissue without direct contact to blood capillaries which are most likely myoepithelial cells or fibroblasts. Moreover, post-EMT glandular epithelial cells might become α-SMA positive as well. However, our study only focused on the CD34/α-SMA double positive cells directly contacting capillary endothelial cells in IR-damaged SGs.

Although the entire role of SG-resident CD34-positive cells remains elusive, it is conceivable that this progenitor cell type could be involved in the healing and the regenerative processes after SG injury by either direct differentiation into vascular and non-vascular stromal cells or by an indirect mechanism, e.g. by secreting micro vesicles and/or soluble factors that can help to (a) induce and sustain the regenerative processes and (b) support revascularization of SG tissue in healing and/or regeneration after injury or pathological damage.

CD34 is predominantly regarded as the characteristic antigen of hematopoietic stem and endothelial progenitor cells (Kwon et al. 2014; Tanaka et al. 2018), and MSCs are thought not to express CD34. However, accumulating recent evidences demonstrate that CD34 is also expressed in freshly isolated MSC fractions from bone marrow and adipose tissue, and that it disappears through the expansion of MSC in culture (Kaiser et al. 2007; Lin et al. 2008; Kuçi et al. 2010; Ferraro et al. 2013). Furthermore, CD34 is considered to be expressed in tissue-resident MSCs (Lin et al. 2012) and microarray analyses revealed that these cells express genes that are known to be involved in vasculogenesis and angiogenesis (Copland et al. 2008). Interestingly, Campagnolo et al. reported that CD34^+^CD31^−^ cells, in human saphenous veins contain a population of MSCs which promote neovascularization by physical and paracrine interaction with endothelial cells (Campagnolo et al. 2010). Here, we show, that a portion of SG-resident CD34-positive cells co-express MSC-associated markers such as Sca-1, c-Kit and CD90. However, they are consistently negative for CD31 after IR, which indicates that they are not progenitors which deliver endothelial cells during SG tissue regeneration. In summary, our data suggest that SG-resident CD34-positive cells putatively represent a sub-population of MSCs, which possess the plasticity to give rise to pericytes and smooth muscle cells and probably some other types of SG stromal cells and by this play a key role for homeostatic maintenance of SG tissue vascularization after radiation-induced injury. Some reports show that MSCs possess a better antioxidant reactive oxygen species-scavenging capacity and active double-strand break repair which increases their radioresistance (Chen et al. 2006; He et al. 2019). We hypothesize that vascular wall resident CD34-positive cells might resist radiation via similar mechanisms. The number of SG-resident CD34-positive cells did not decline following IR and even increased temporarily. This suggests that SG-resident CD34-positive cells proliferate and/or reside in the vascular adventitia from where they can be mobilized and activated during tissue healing. 20 weeks after IR, the number of SG-resident CD34-positive cells gradually declined and returned to the level before IR. It has been suggested that CD34 expression correlates with replicative capacity and stemness, and loss of CD34 expression might be related to lineage commitment (Kaiser et al. 2007; Suga et al. 2009). We hypothesize that the regenerative processes after IR go alongside with an expansion of the stem/progenitor cell pool. After the healing phase, the number of CD34-positive cells return to the level before IR.

Communication between MSC and endothelial cells (EC) is recognized as important cellular interaction in angiogenesis. However, the underlying mechanisms of this biological processes are not well understood. Xue et al. (2013) reported that co-culturing ECs with MSCs induces TGF-β signaling and an up-regulation of genes related to angiogenesis such as platelet/endothelial cell adhesion molecule-1 (PECAM-1), cadherin 5 (CDH5), and angiopoietin-related protein 4 (ANGPTL4). These findings demonstrate that the crosstalk between ECs and MSCs has a significant impact on angiogenesis. In our report, we could observe that resident-CD34 positive cells (MSC like phenotype) enwrap endothelial sprouts from the outside, probably supporting angiogenesis and the maturation of blood vessels. We performed initial experiments demonstrating that *tgf-β, pecam-1, cdh5, angptl4* gene expression was increased in injured SGs after IR (Suppl. Fig. 1). Although, the most mechanisms of interaction between MSCs and ECs in angiogenesis are unknown, we assume that CD34 positive cells (MSC like phenotype) and CD31 positive cells (ECs) might communicate via TGF-β signaling. TGF-ß is also one of the key factors that induce endothelial-to-mesenchymal transition (EndMT) during which endothelial cells acquire a mesenchymal phenotype. We cannot exclude EndMT in processes after IR, though we did not observe a particular switch of the mature endothelial cells into an MSC phenotype. Therefore, our initial findings need to be further investigated in more detail in future studies.

In conclusion, our results demonstrate that SG-resident CD34-positive cells show a MSC-like phenotype and survive and even expand after severe IR-induced SG damage. Moreover, they contribute to new vessel formation and/or remodeling of impaired capillaries within the parenchyma of SG. However, the exact mechanism of resident CD34-postive cell migration and differentiation remains largely unknown. Therefore, additional future studies are worth to be done to use the potential of these progenitor cells therapeutically in healing of SG and particularly in radiation-induced xerostomia.

## Electronic supplementary material

Below is the link to the electronic supplementary material.

Supplemental Figure 1 The mRNA expressions of il-1β, tgf-β, pecam-1, cdh5, angptl4, and aqp5 genes in the submandibular glands at 8 weeks post-IR compared to non-irradiated mice. The mRNA expression of il-1β, tgf-β, pecam-1, cdh5, and angptl4 were significantly up-regulated in irradiated submandibular glands (*p<0.05). Meanwhile, aqp5 mRNA expression was significantly down-regulated in irradiated submandibular glands compared to non-irradiated mice (*p<0.05). Experimental values are presented as mean values of normalized RNA expression ±SD from 3 independent experiments and n=3 in each group

Supplemental Figure 2 Changes of salivary flow rate (SFR) at 0, 4, 8, and 12 weeks after IR (**p < 0.01) compared to non-irradiated mice. SFR was determined at week 0, 4, 8, and 12 post-IR. Experimental values are presented as mean values ±SD: n=4 in each group at each time points

Supplemental Figure 3 Paraffin-sections of submandibular glands with no IR (A), at 4-weeks (B and C), and at 20-weeks (D) after IR were stained for CD34. Sections were counterstained with Nuclear Fast Red. Resident CD34-positive cells locate in the connective tissues (not in acini or duct) and around the blood vessels. Scale bar; 50 µm. BV; Blood Vessel, a; acini, d; duct

Supplemental Figure 4 Triple immunofluorescence staining for CD31 (Red), CD34 (Purple) and α-SMA (α-smooth muscle actin) (Green) in submandibular gland with no IR (A) and at 1-week (B), 4-weeks (C), 8-weeks (D), and 20-weeks (E) after IR. Scale bar; 50 µm. Blue; DAPI, Green; α-SMA, Purple; CD34, Red; CD31

Supplemental Figure 5 Changes of the number of Ki-67 positive cells at 1-, 3-, and 7- days after IR. Asterisk represents statistical significance compared with no irradiated submandibular glands (**p < 0.01)

Supplemental Figure 6 Double immunofluorescence staining for CD34 (Green) and CD31 (Red) in parenchymal of submandibular gland with no IR (A) and at 20-weeks after IR (B). Scale bar; 10 µm. Blue; DAPI, Green; CD34, Red; CD31

## Abbreviations

CD: Cluster of differentiation/cluster designation
CXCR1: CXC chemokine receptor 1
IR: Irradiation
SFR: Salivary flow rate
SG: Salivary gland
MSC: Mesenchymal stem/stromal cell
3D-CRT: 3-Dimensional conformal radiotherapy
IMRT: Intensity modulated radiation therapy
α-SMA: α-Smooth muscle actin
ip: Intraperitoneal
PFA: Paraformaldehyde
H&E: Hematoxylin and eosin
DAPI: 4′,6-Diamidino-2-phenylindole
IL-1β: Interleukin-1β
TGF-β: Transforming growth factor-β
PECAM-1: Platelet endothelial cell adhesion molecule
CDH5: Cadherin 5
ANGPTL4: Angiopoietin-like 4
AQP5: Aquaporin 5
CEACAM1: Carcinoembryonic antigen-related cell adhesion molecule 1
TEM: Transmission electron microscope
EMT: Epithelial-mesenchymal transition
ECM: Extracellular matrix
KDR: Kinase insert domain receptor
HITA: Human internal thoracic artery

## References

[CR1] Alessandri G, Girelli M, Taccagni G, Colombo A, Nicosia R, Caruso A, Baronio M, Pagano S, Cova L, Parati E (2001). Human vasculogenesis ex vivo: embryonal aorta as a tool for isolation of endothelial cell progenitors. Lab Invest.

[CR2] Alunno A, Ibba-Manneschi L, Bistoni O, Rosa I, Caterbi S, Gerli R, Manetti M (2015). Telocytesin minor salivary glands of primary Sjögren's syndrome: association with the extent of inflammation and ectopic lymphoid neogenesis. J Cell Mol Med.

[CR3] Baum BJ, Zheng C, Cotrim AP, McCullagh L, Goldsmith CM, Brahim JS, Atkinson JC, Turner RJ, Liu S, Nikolov N, Illei GG (2009). Aquaporin-1 gene transfer to correct radiation-induced salivary hypofunction. Handb Exp Pharmacol.

[CR4] Bourguignon LY, Shiina M, Li JJ (2014). Hyaluronan-CD44 interaction promotes oncogenic signaling, microRNA functions, chemoresistance, and radiation resistance in cancer stem cells leading to tumor progression. Adv Cancer Res.

[CR5] Braam PM, Terhaard CH, Roesink JM, Raaijmakers CP (2006). Intensity-modulated radiotherapy significantly reduces xerostomia compared with conventional radiotherapy. Int J Radiat Oncol Biol Phys.

[CR6] Chen MF, Lin CT, Chen WC, Yang CT, Chen CC, Liao SK, Liu JM, Lu CH, Lee KD (2006). The sensitivity of human mesenchymal stem cells to ionizing radiation. Int J Radiat Oncol Biol Phys.

[CR7] Cichy J, Puré E (2003). The liberation of CD44. J Cell Biol.

[CR8] Campagnolo P, Cesselli D, Al Haj Zen A, Beltrami AP, Kränkel N, Katare R, Angelini G, Emanueli C, Madeddu P (2010). Human adult vena saphena contains perivascular progenitor cells endowed with clonogenic and proangiogenic potential. Circulation.

[CR9] Copland I, Sharma K, Lejeune L, Eliopoulos N, Stewart D, Liu P, Lachapelle K, Galipeau J (2008). CD34 expression on murine marrow-derived mesenchymal stromal cells: impact on neovascularization. Exp Hematol.

[CR10] Culemann S, Grüneboom A, Nicolás-Ávila JÁ, Weidner D, Lämmle KF, Rothe T, Quintana JA, Kirchner P, Krljanac B, Eberhardt M, Ferrazzi F, Kretzschmar E, Schicht M, Fischer K, Gelse K, Faas M, Pfeifle R, Ackermann JA, Pachowsky M, Renner N, Simon D, Has-eloff RF, Ekici AB, Bäuerle T, Blasig IE, Vera J, Voehringer D, Kleyer A, Paulsen F, Schett G, Hidalgo A, Krönke G (2019). Locally renewing resident synovial macrophagesprovide a protective barrier for the joint. Nature.

[CR11] Dick SA, Zaman R, Epelman S (2019). Using high-dimensional approaches to probe monocytes and macrophages in cardiovascular disease. Front Immunol.

[CR12] Dirix P, Nuyts S, Van den Bogaert W (2006). Radiation-induced xerostomia in patients with head and neck cancer: a literature review. Cancer.

[CR13] Ferraro GA, De Francesco F, Nicoletti G, Paino F, Desiderio V, Tirino V, D'Andrea F (2013). Human adipose CD34+ CD90+ stem cells and collagen scaffold constructs grafted in vivo fabricate loose connective and adipose tissues. J Cell Biochem.

[CR14] Grundmann O, Mitchell GC, Limesand KH (2009). Sensitivity of salivary glands to radiation: from animal models to therapies. J Dent Res.

[CR15] Hall BE, Zheng C, Swaim WD, Cho A, Nagineni CN, Eckhaus MA, Flanders KC, Ambudkar IS, Baum BJ, Kulkarni AB (2010). Conditional overexpression of TGF-beta1 disrupts mouse salivary gland development and function. Lab Investig.

[CR16] Hatton CF, Duncan CJA (2019). Microglia are essential to protective antiviral immunity: lessons from mouse models of viral encephalitis. Front Immunol.

[CR17] He N, Xiao C, Sun Y, Wang Y, Du L, Feng Y, Liu Y, Wang Q, Ji K, Wang J, Zhang M, Xu C, Liu Q (2019). Radiation responses of human mesenchymal stem cells derived from different sources. Dose Response.

[CR18] Takashi I, Sumita Y, Yoshida T, Honma R, Iwatake M, Raudales JLM, Shizuno T, Kuroshima S, Masuda H, Seki M, Tran SD, Asahara T, Asahina I (2019). Anti-inflammatory and vasculogenic conditioning of peripheral blood mononuclear cells reinforces their therapeutic potential for radiation-injured salivary glands. Stem Cell Res Ther.

[CR345] Invernici G, Emanueli C, Madeddu P, Cristini S, Gadau S, Benetti A, Ciusani E, Stassi G, Siragusa M, Nicosia R, Peschle C, Fascio U, Colombo A, Rizzuti T, Parati E, Alessandri G (2007) Human fetal aorta contains vascular progenitor cells capable of inducing vasculogenesis, angiogenesis, and myogenesis in vitro and in a murine model of peripheral ischemia. Am J Pathol 170:1879–1892. 10.2353/ajpath.2007.060646 10.2353/ajpath.2007.060646 PMC1899439 17525256

[CR19] Ingram DA, Mead LE, Moore DB, Woodard W, Fenoglio A, Yoder MC (2005). Vessel wall-derived endothelial cells rapidly proliferate because they contain a complete hierarchy of endothelial progenitor cells. Blood.

[CR20] Isacke CM, Yarwood H (2002). The hyaluronan receptor, CD44. Int J Biochem Cell Biol.

[CR21] Kaiser S, Hackanson B, Follo M, Mehlhorn A, Geiger K, Ihorst G, Kapp U (2007). BM cells giving rise to MSC in culture have a heterogeneous CD34 and CD45 phenotype. Cytotherapy.

[CR22] Klein D, Hohn HP, Kleff V, Tilki D, Ergün S (2010). Vascular wall-resident stem cells. Histol Histopathol.

[CR346] Klein D, Weisshardt P, Kleff V, Jastrow H, Jakob HG, Ergün S (2011) Vascular wall-resident CD44+ multipotent stem cells give rise to pericytes and smooth muscle cells and contribute to new vessel maturation. PLoS One 6(5):e20540. 10.1371/journal.pone.0020540 10.1371/journal.pone.0020540 PMC3102739 21637782

[CR23] Klein D, Meissner N, Kleff V, Jastrow H, Yamaguchi M, Ergün S, Jendrossek V (2014). Nestin(+) tissue-resident multipotent stem cells contribute to tumor progression by differentiating into pericytes and smooth muscle cells resulting in blood vessel remodeling. Front Oncol.

[CR24] Kosmac K, Peck BD, Walton RG, Mula J, Kern PA, Bamman MM, Dennis RA, Jacobs CA, Lattermann C, Johnson DL, Peterson CA (2018). Immunohistochemical identification of human skeletal muscle macrophages. Bio Protoc.

[CR25] Kramann R, Goettsch C, Wongboonsin J, Iwata H, Schneider RK, Kuppe C, Kaesler N, Chang-Panesso M, Machado FG, Gratwohl S, Madhurima K, Hutcheson JD, Jain S, Aikawa E, Humphreys BD (2016). Adventitial MSC-like cells are progenitors of vascular smooth muscle cells and drive vascular calcification in chronic kidney disease. Cell Stem Cell.

[CR26] Kuçi S, Kuçi Z, Kreyenberg H, Deak E, Pütsch K, Huenecke S, Amara C, Koller S, Rettinger E, Grez M, Koehl U, Latifi-Pupovci H, Henschler R, Tonn T, von Laer D, Klingebiel T, Bader P (2010). CD271 antigen defines a subset of multipotent stromal cells with immunosuppressive and lymphohematopoietic engraftment-promoting properties. Haematologica.

[CR347] Kwon SM, Lee JH, Lee SH, Jung SY, Kim DY, Kang SH, Yoo SY, Hong JK, Park JH, Kim JH, Kim SW, Kim YJ, Lee SJ, Kim HG, Asahara T (2014) Cross talk with hematopoietic cells regulates the endothelial progenitor cell differentiation of CD34 positive cells. PLoS One 9(8):e106310. 10.1371/journal.pone.0106310 10.1371/journal.pone.0106310 PMC4148437 25166961

[CR27] Lim JY, Ra JC, Shin IS, Jang YH, An HY, Choi JS, Kim WC, Kim YM (2013). Systemic trans-plantation of human adipose tissue-derived mesenchymal stem cells for the regeneration of irradiation-induced salivary gland damage. PLoS ONE.

[CR28] Lin CS, Ning H, Lin G, Lue TF (2012). Is CD34 truly a negative marker for mesenchymal stromal cells?. Cytotherapy.

[CR29] Lin G, Garcia M, Ning H, Banie L, Guo YL, Lue TF, Lin CS (2008). Defining stem and progenitor cells within adipose tissue. Stem Cells Dev.

[CR30] Malouf JG, Aragon C, Henson BS, Eisbruch A, Ship JA (2003). Influence of parotid-sparing radiotherapy on xerostomia in head and neck cancer patients. Cancer Detect Prev.

[CR31] Manetti M, Tani A, Rosa I, Chellini F, Squecco R, Idrizaj E, Zecchi-Orlandini S, Ibba-Manneschi L, Sassoli C (2019). Morphological evidence for telocytes as stromal cells supporting satellite cell activation in eccentric contraction-induced skeletal muscle injury. Sci Rep.

[CR32] Maria OM, Maria O, Liu Y, Komarova SV, Tran SD (2011). Matrigel improves functional proper-ties of human submandibular salivary gland cell line. Int J Biochem Cell Biol.

[CR33] Maria OM, Maria AM, Cai Y, Tran SD (2012). Cell surface markers CD44 and CD166 localized specific populations of salivary acinar cells. Oral Dis.

[CR34] Matsuzaki T, Susa T, Shimizu K, Sawai N, Suzuki T, Aoki T, Yokoo S, Takata K (2012). Function of the membrane water channel aquaporin-5 in the salivary gland. Acta Histochem Cytochem.

[CR35] McGonagle D, Baboolal TG, Jones E (2017). Native joint-resident mesenchymal stem cells for cartilage repair in osteoarthritis. Nat Rev Rheumatol.

[CR36] Mekala SR, Wörsdörfer P, Bauer J, Stoll O, Wagner N, Reeh L, Loew K, Eckner G, Kwok CK, Wischmeyer E, Dickinson ME, Schulze H, Stegner D, Benndorf RA, Edenhofer F, Pfeiffer V, Kuerten S, Frantz S, Ergün S (2018). Generation of cardiomyocytes from vascular adventitia-resident stem cells. Circ Res.

[CR37] Mizrachi A, Cotrim AP, Katabi N, Mitchell JB, Verheij M, Haimovitz-Friedman A (2016). Radiation-induced microvascular injury as a mechanism of salivary gland hypofunction and potential target for radio protectors. Radiat Res.

[CR38] Nguyen T, Chen CJ, Shively JE (2014). Phosphorylation of CEACAM1 molecule by calmodulin kinase IID in a three-dimensional model of mammary gland lumen formation. J Biol Chem.

[CR39] Passman JN, Dong XR, Wu SP, Maguire CT, Hogan KA, Bautch VL, Majesky MW (2008). A sonic hedgehog signaling domain in the arterial adventitia supports resident Sca1+ smooth muscle progenitor cells. Proc Natl Acad Sci USA.

[CR40] Ponta H, Sherman L, Herrlich PA (2003). CD44: from adhesion molecules to signalling regulators. Nat Rev Mol Cell Biol.

[CR41] Psaltis PJ, Harbuzariu A, Delacroix S, Holroyd EW, Simari RD (2011). Resident vascular progenitor cells-diverse origins, phenotype, and function. J Cardiovasc Transl Res.

[CR42] Psaltis PJ, Harbuzariu A, Delacroix S, Witt TA, Holroyd EW, Spoon DB, Hoffman SJ, Pan S, Kleppe LS, Mueske CS, Gulati R, Sandhu GS, Simari RD (2012). Identification of a monocyte-predisposed hierarchy of hematopoietic progenitor cells in the adventitia of postnatal murine aorta. Circulation.

[CR43] Psaltis PJ, Simari RD (2015). Vascular wall progenitor cells in health and disease. Circ Res.

[CR44] Radfar L, Sirois DA (2003). Structural and functional injury in minipig salivary glands following fractionated exposure to 70 Gy of ionizing radiation: an animal model for human radiation-induced salivary gland injury. Oral Surg Oral Med Oral Pathol Oral Radiol Endod.

[CR45] Rahmani W, Liu Y, Rosin NL, Kline A, Raharjo E, Yoon J, Stratton JA, Sinha S, Biernaskie J (2018). Macrophages promote wound-induced hair follicle regeneration in a CX3CR1-and TGF-β1-dependent manner. J Invest Dermatol.

[CR46] Rueckschloss U, Kuerten S, Ergün S (2016). The role of CEA-related cell adhesion molecule-1 (CEACAM1) in vascular homeostasis. Histochem Cell Biol.

[CR47] Sanjurjo-Rodriguez C, Baboolal TG, Burska AN, Ponchel F, El-Jawhari JJ, Pandit H, McGonagle D, Jones E (2019). Gene expression and functional comparison between multipotential stromal cells from lateral and medial condyles of knee osteoarthritis patients. Sci Rep.

[CR48] Santamaria-Barria JA, Zeng S, Greer JB, Beckman MJ, Seifert AM, Cohen NA, Zhang JQ, Crawley MH, Green BL, Loo JK, Maltbaek JH, DeMatteo RP (2019). Csf1r or Mer inhibition delays liver regeneration via suppression of Kupffer cells. PLoS ONE.

[CR49] Seiwert TY, Cohen EE (2005). State-of-the-art management of locally advanced head and neck cancer. Br J Cancer.

[CR50] Sisto M, Lisi S, Ribatti D (2018). The role of the epithelial-to-mesenchymal transition (EMT) in diseases of the salivary glands. Histochem Cell Biol.

[CR51] St John MA, Abemayor E, Wong DT (2006). Recent new approaches to the treatment of head and neck cancer. Anticancer Drugs.

[CR52] Suga H, Matsumoto D, Eto H, Inoue K, Aoi N, Kato H, Araki J, Yoshimura K (2009). Functional implications of CD34 expression in human adipose-derived stem/progenitor cells. Stem Cells Dev.

[CR53] Sumita Y, Liu Y, Khalili S, Maria OM, Xia D, Key S, Cotrim AP, Mezey E, Tran SD (2011). Bone marrow-derived cells rescue salivary gland function in mice with head and neck irradiation. Int J Biochem Cell Biol.

[CR54] Takeyama A, Yoshikawa Y, Ikeo T, Morita S, Hieda Y (2015). Expression patterns of CD66a andCD117 in the mouse submandibular gland. Acta Histochem.

[CR55] Tanaka J, Ogawa M, Hojo H, Kawashima Y, Mabuchi Y, Hata K, Nakamura S, Yasuhara R, Takamatsu K, Irié T, Fukada T, Sakai T, Inoue T, Nishimura R, Ohara O, Saito I, Ohba S, Tsuji T, Mishima K (2018). Generation of orthotopically functional salivary gland fr-om embryonic stem cells. Nat Commun.

[CR56] Tintut Y, Alfonso Z, Saini T, Radcliff K, Watson K, Boström K, Demer LL (2003). Multilineage potential of cells from the artery wall. Circulation.

[CR57] Togarrati PP, Sasaki RT, Abdel-Mohsen M, Dinglasan N, Deng X, Desai S, Emmerson E, Yee E, Ryan WR, da Silva MCP, Knox SM, Pillai SK, Muench MO (2017). Identification and characterization of a rich population of CD34+ mesenchymal stem/stromal cells in hum-an parotid, sublingual and submandibular glands. Sci Rep.

[CR58] Trotti A, Bellm LA, Epstein JB, Frame D, Fuchs HJ, Gwede CK, Komaroff E, Nalysnyk L, Zilberberg MD (2003). Mucositis incidence, severity and associated outcomes in patients with head and neck cancer receiving radiotherapy with or without chemotherapy: a systematic literature review. Radiother Oncol.

[CR59] Weng C, Nguyen T, Shively JE (2016). miRNA-342 Regulates CEACAM1-induced lumen formation in a three-dimensional model of mammary gland morphogenesis. J Biol Chem.

[CR60] Wu VWC, Leung KY (2019). A review on the assessment of radiation induced salivary gland damage after radiotherapy. Front Oncol.

[CR61] Wörsdörfer P, Mekala SR, Bauer J, Edenhofer F, Kuerten S, Ergün S (2017). The vascular adventitia: an endogenous, omnipresent source of stem cells in the body. Pharmacol Ther.

[CR62] Yoshikawa M, Morine Y, Ikemoto T, Imura S, Higashijima J, Iwahashi S, Saito YU, Takasu C, Yamada S, Ishikawa D, Teraoku H, Takata A, Yoshimoto T, Shimada M (2017). Elevated preoperative serum CEA level is associated with poor prognosis in patients with hepatocellular carcinoma through the epithelial-mesenchymal transition. Anticancer Res.

[CR63] Xue Y, Xing Z, Bolstad AI, Van Dyke TE, Mustafa K (2013). Co-culture of human bone marrow stromal cells with endothelial cells alters gene expression profiles. Int J Artif Organs.

[CR64] Zengin E, Chalajour F, Gehling UM, Ito WD, Treede H, Lauke H, Weil J, Reichenspurner H, Kilic N, Ergün S (2006). Vascular wall resident progenitor cells: a source for postnatal vasculogenesis. Development.

[CR65] Zhang J, Cui L, Xu M, Zheng Y (2014). Restoring the secretory function of irradiation-damaged salivary gland by administrating deferoxamine in mice. PLoS ONE.

