# Supplementary figures and images for: Resident CD34-positive cells contribute to peri-endothelial cells and vascular morphogenesis in salivary gland after irradiation
Source: J Neural Transm (Vienna). 2020 Oct 6;127(11):1467–79. doi: 10.1007/s00702-020-02256-1 (PMC7578140; doi:10.1007/s00702-020-02256-1)

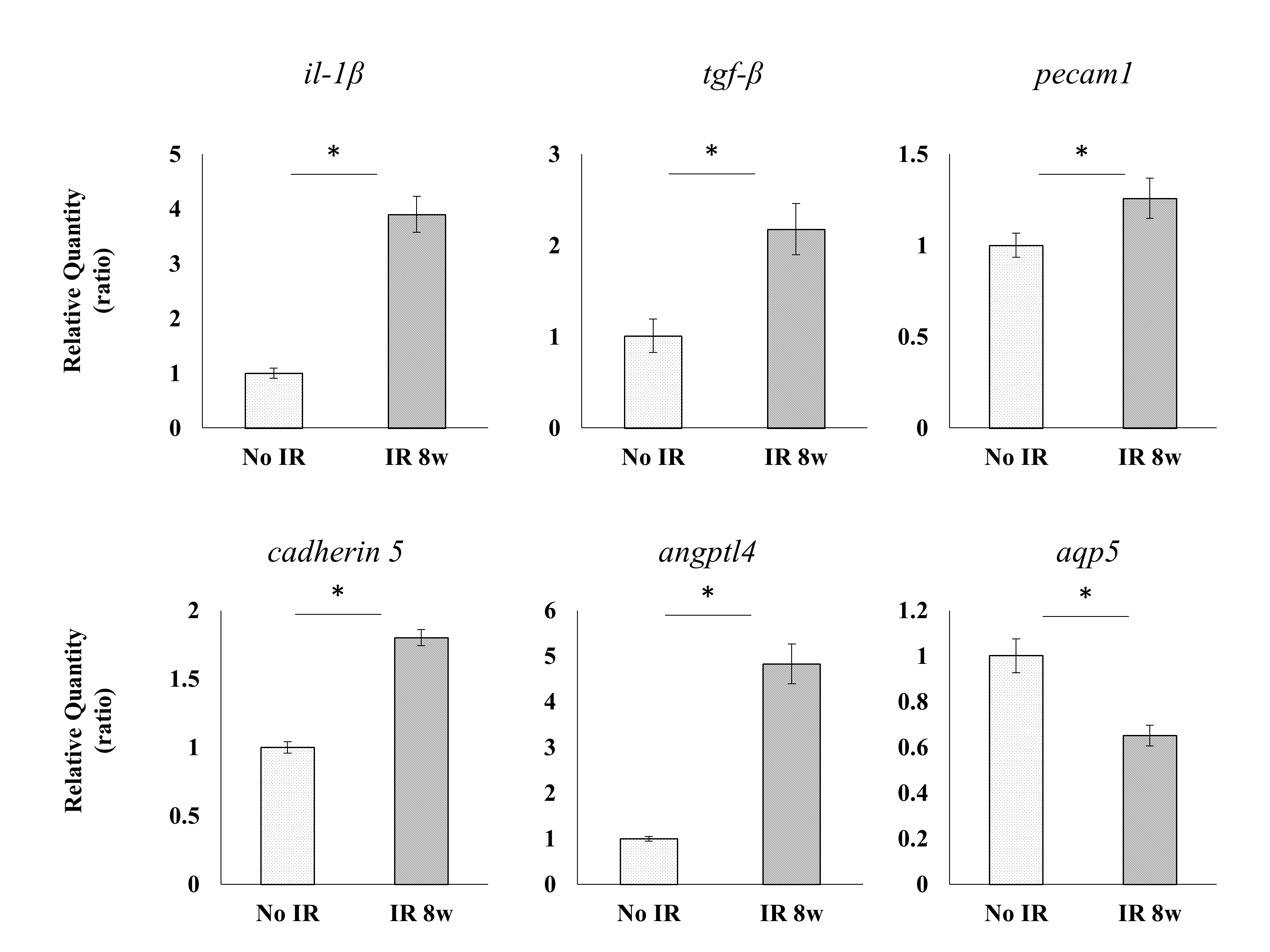

Supplement: Supplementary file 1 — Supplemental Figure 1 The mRNA expressions of il-1β, tgf-β, pecam-1, cdh5, angptl4, and aqp5 genes in the submandibular glands at 8 weeks post-IR compared to non-irradiated mice. The mRNA expression of il-1β, tgf-β, pecam-1, cdh5, and angptl4 were significantly up-regulated in irradiated submandibular glands (*p<0.05). Meanwhile, aqp5 mRNA expression was significantly down-regulated in irradiated submandibular glands compared to non-irradiated mice (*p<0.05). Experimental values are presented as mean values of normalized RNA expression ±SD from 3 independent experiments and n=3 in each group [file 702_2020_2256_MOESM1_ESM.tif]

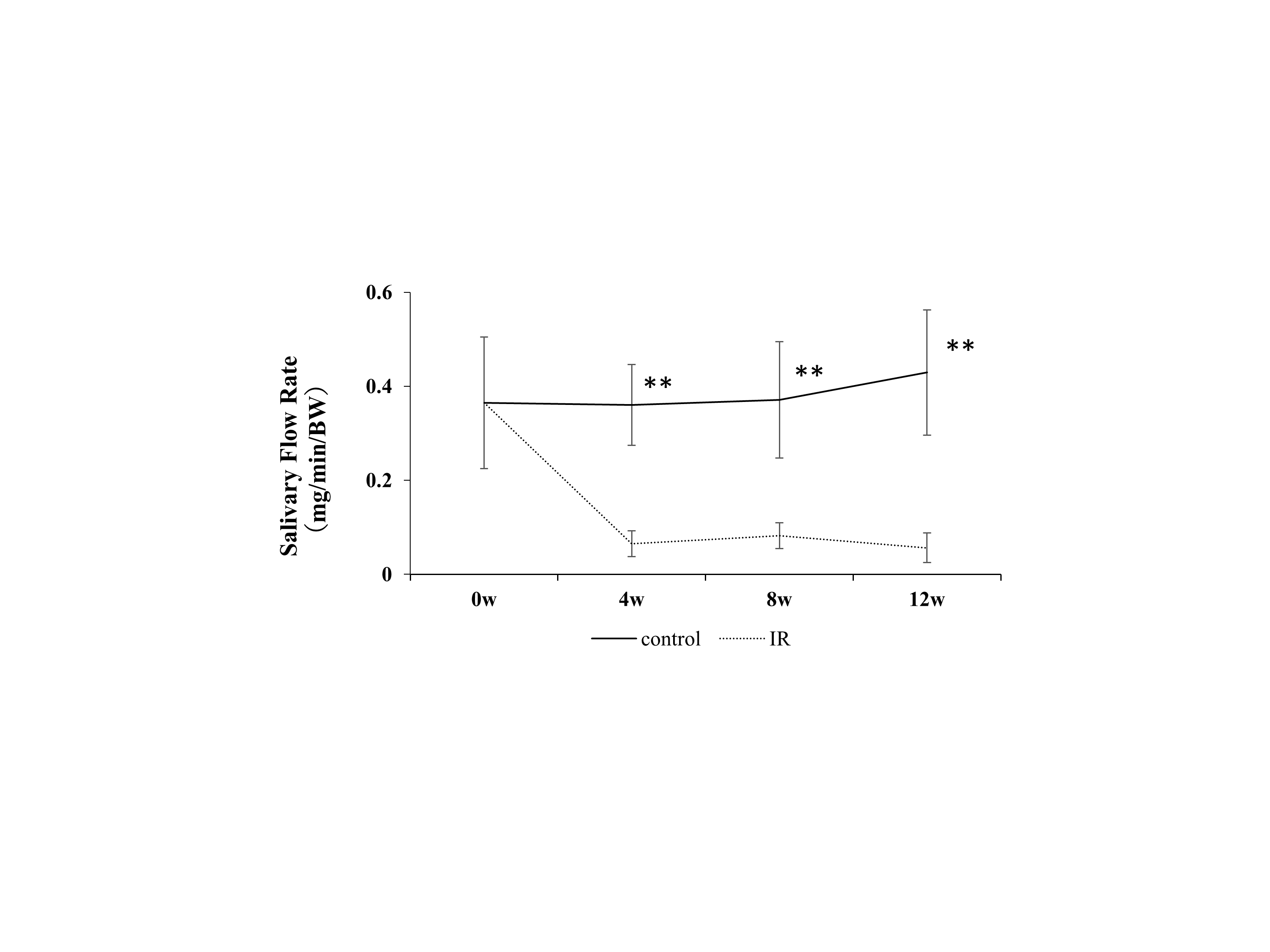

Supplement: Supplementary file 2 — Supplemental Figure 2 Changes of salivary flow rate (SFR) at 0, 4, 8, and 12 weeks after IR (**p < 0.01) compared to non-irradiated mice. SFR was determined at week 0, 4, 8, and 12 post-IR. Experimental values are presented as mean values ±SD: n=4 in each group at each time points [file 702_2020_2256_MOESM2_ESM.tif]

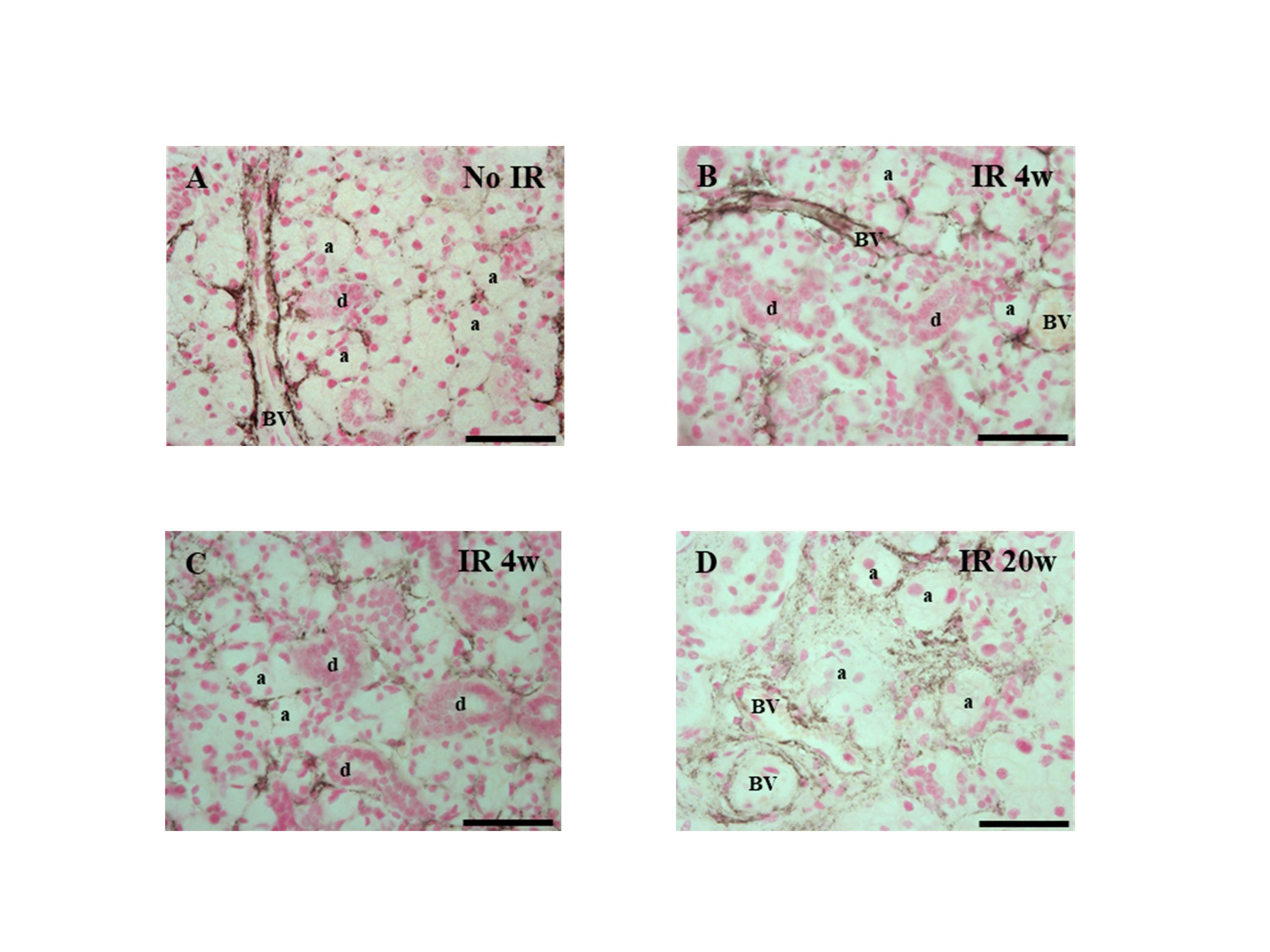

Supplement: Supplementary file 3 — Supplemental Figure 3 Paraffin-sections of submandibular glands with no IR (A), at 4-weeks (B and C), and at 20-weeks (D) after IR were stained for CD34. Sections were counterstained with Nuclear Fast Red. Resident CD34-positive cells locate in the connective tissues (not in acini or duct) and around the blood vessels. Scale bar; 50 µm. BV; Blood Vessel, a; acini, d; duct [file 702_2020_2256_MOESM3_ESM.tif]

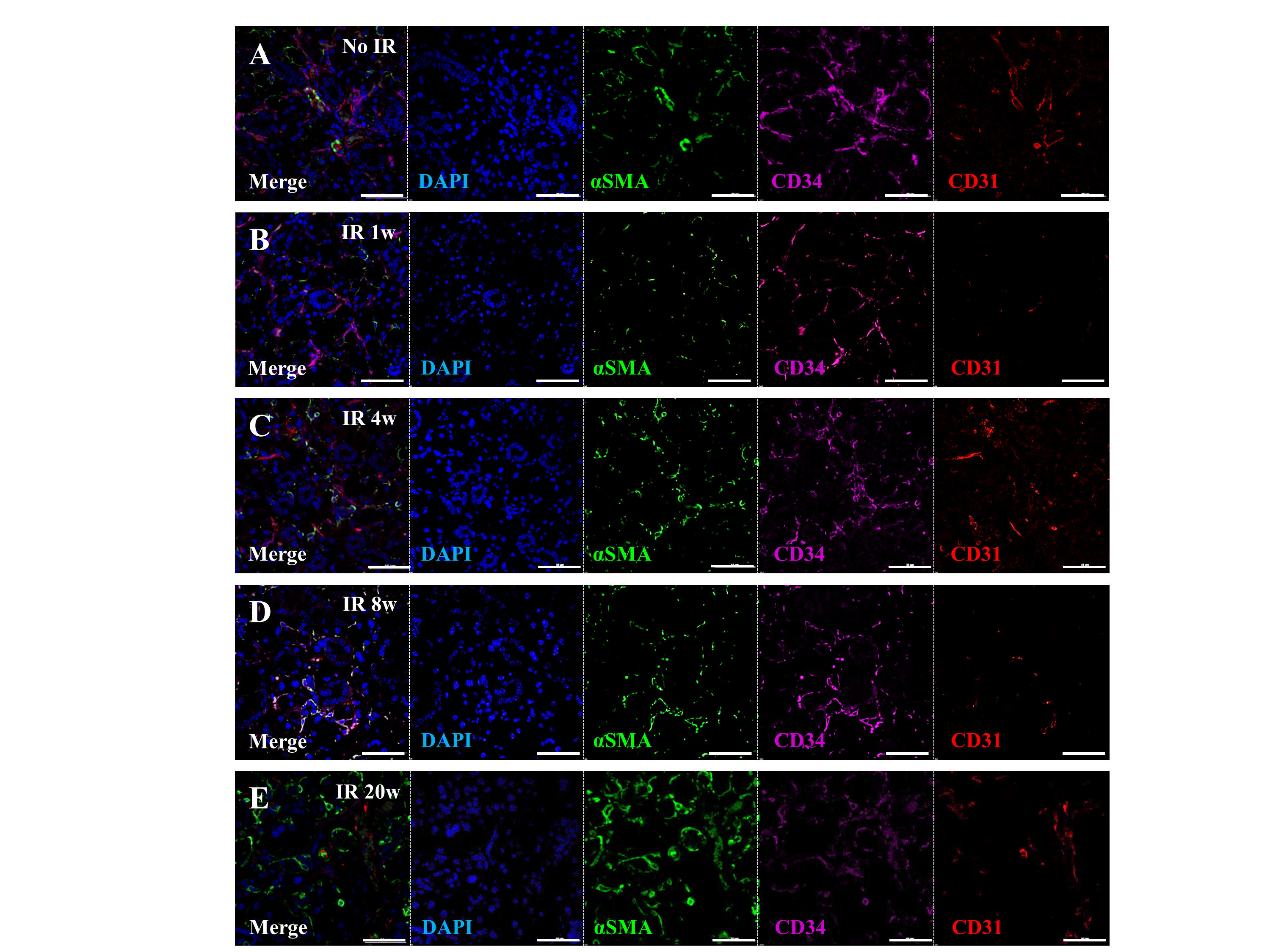

Supplement: Supplementary file 4 — Supplemental Figure 4 Triple immunofluorescence staining for CD31 (Red), CD34 (Purple) and α-SMA (α-smooth muscle actin) (Green) in submandibular gland with no IR (A) and at 1-week (B), 4-weeks (C), 8-weeks (D), and 20-weeks (E) after IR. Scale bar; 50 µm. Blue; DAPI, Green; α-SMA, Purple; CD34, Red; CD31 [file 702_2020_2256_MOESM4_ESM.tif]

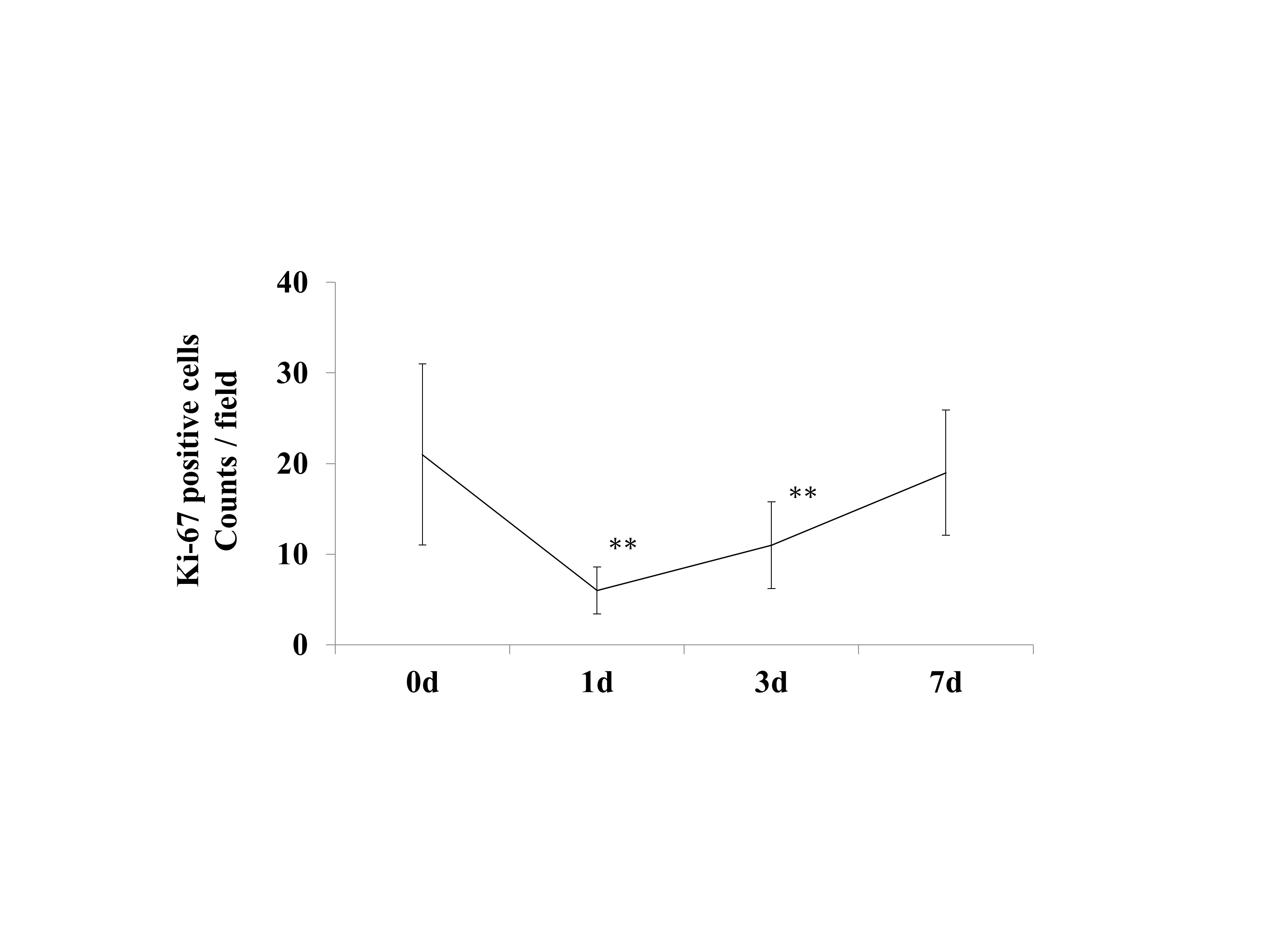

Supplement: Supplementary file 5 — Supplemental Figure 5 Changes of the number of Ki-67 positive cells at 1-, 3-, and 7- days after IR. Asterisk represents statistical significance compared with no irradiated submandibular glands (**p < 0.01) [file 702_2020_2256_MOESM5_ESM.tif]

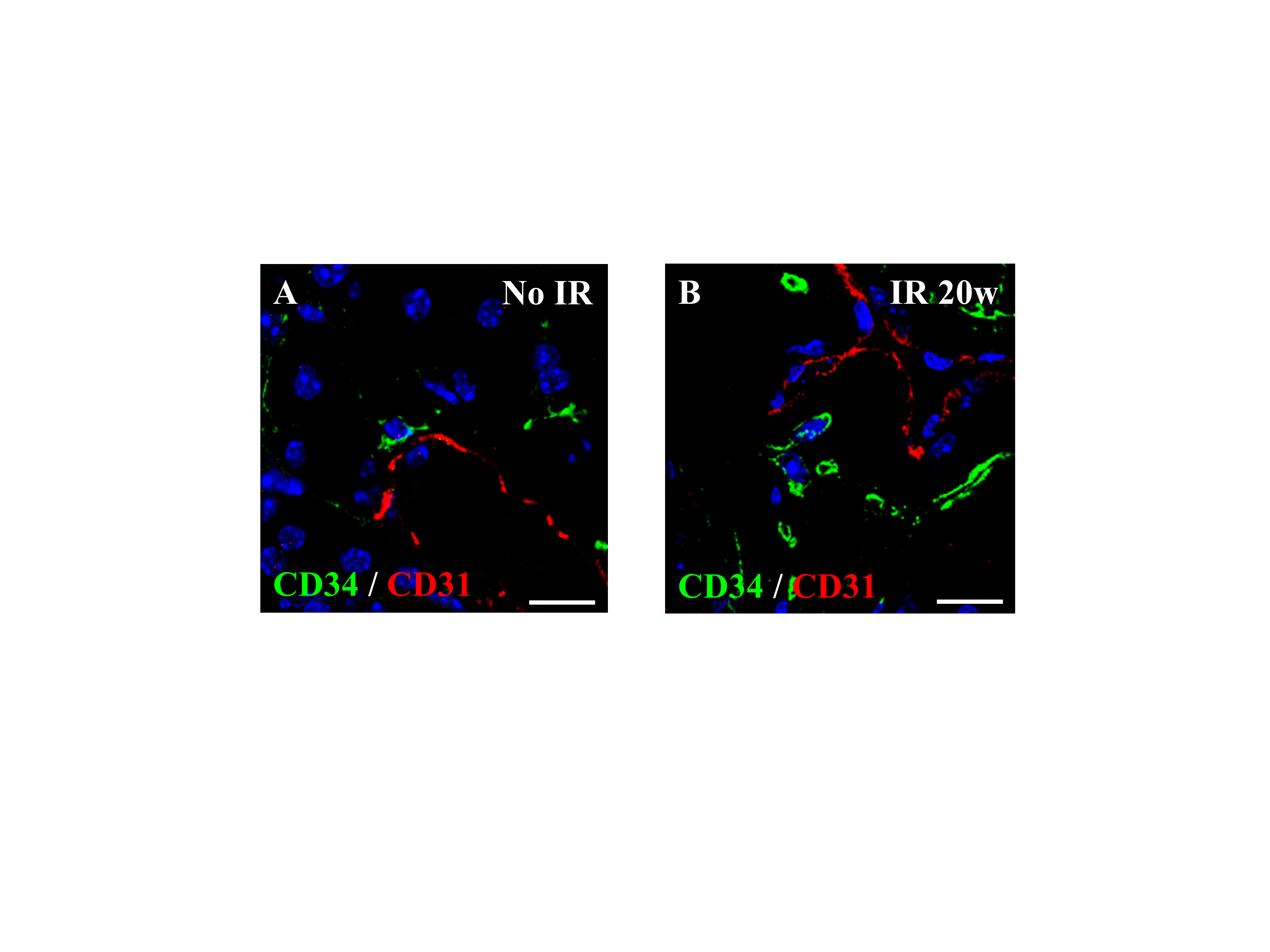

Supplement: Supplementary file 6 — Supplemental Figure 6 Double immunofluorescence staining for CD34 (Green) and CD31 (Red) in parenchymal of submandibular gland with no IR (A) and at 20-weeks after IR (B). Scale bar; 10 µm. Blue; DAPI, Green; CD34, Red; CD31 [file 702_2020_2256_MOESM6_ESM.tif]
